# Attack rate reductions following berotralstat initiation among US patients with hereditary angioedema in the real-world

**DOI:** 10.1186/s13223-025-01005-x

**Published:** 2026-01-25

**Authors:** Mark Davis-Lorton, Raffi Tachdjian, Lorena Lopez-Gonzalez, Sean D. MacKnight, Ramya Ramasubramanian, François Laliberté, Patrick Gillard, Meri LiVecchi, Sandra Nestler-Parr, William R. Lumry

**Affiliations:** 1 ENT and Allergy Associates, Tarrytown, NY USA; 2 Department of Pediatrics, University of California Los Angeles, 1301 20th Street, Suite 380, Los Angeles, Santa Monica, CA 90404 USA; 3 BioCryst Pharmaceuticals, Inc., Durham, NC USA; 4 Groupe d’analyse SRI, Montreal, QC Canada; 5 Analysis Group, Inc., Los Angeles, CA USA; 6 Allergy and Asthma Research Associates, Dallas, TX USA

**Keywords:** Hereditary angioedema, Berotralstat, Long-term prophylaxis, Rare disease, Real-world evidence

## Abstract

**Background:**

Hereditary angioedema (HAE) causes recurring swelling attacks, leading to substantial disease burden. This real-world, retrospective study aimed to evaluate HAE attack rates before and after berotralstat initiation stratified by patients’ baseline attack frequency.

**Methods:**

Specialty Pharmacy data from Optime Care, Inc. (12/2020–01/2024), the sole berotralstat dispenser in the United States, were analyzed. Eligible patients had  ≥ 2 berotralstat dispensings (first =  index) and ≥ 1 self-assessment of attacks at baseline (90-days pre-index) and follow-up (first-to-last dispensing). Patients were classified by HAE type (based on laboratory measurements) and baseline attacks (≥ 5, 2–4, 1, and 0 attacks/month). Follow-up attack rates were compared with baseline using mean differences, confidence intervals, and* P*-values. Among those with 0 baseline attacks/month, proportions with 0 follow-up attacks/month were assessed.

**Results:**

Of 390 eligible patients with HAE with C1 esterase inhibitor (C1INH) deficiency (HAE-C1INH) and 311 with HAE with normal C1INH (HAE-nC1INH), most were female (64.1% and 77.5%) with mean ages of 39.3 and 48.1 years, respectively. Mean attack rates decreased from 2.50 to 0.79 attacks/month (HAE-C1INH) and from 4.59 to 1.68 attacks/month (HAE-nC1INH) at 12-months of berotralstat treatment (both *P* < 0 .001), with sustained reductions at 18-months. Patients with ≥ 1 baseline attack/month experienced significantly lower attack rates after berotralstat initiation. Among patients with 0 baseline attacks/month, most also maintained 0 attacks/month in each follow-up interval (HAE-C1INH: 70–85%; HAE-nC1INH: 61–81%).

**Conclusion:**

Berotralstat was associated with significant and sustained reductions in attack rates among patients with HAE, regardless of baseline attack rate. Patients with 0 baseline attacks/month maintained low attack rates following berotralstat initiation.

**Supplementary Information:**

The online version contains supplementary material available at 10.1186/s13223-025-01005-x.

## Plain language summary

Hereditary angioedema (HAE) is a rare genetic condition characterized by sudden episodes of swelling in various parts of the body. The swelling can be extremely painful and interrupt daily activities when it occurs in areas such as hands, feet, intestines, or genitals, and potentially fatal in cases of airway swelling. Berotralstat is an oral medication that can prevent HAE attacks. In this study, people diagnosed and living with HAE had fewer HAE attacks after starting berotralstat. When they were grouped based on the frequency of HAE attacks they had before starting treatment, berotralstat decreased the rate of HAE attacks in all groups. Our research supports that berotralstat treatment helps to reduce HAE attacks and should be considered as a treatment option regardless of the number of HAE attacks people are experiencing.

**Supplementary Information:**

The online version contains supplementary material available at 10.1186/s13223-025-01005-x.

## Background

Hereditary angioedema (HAE) is a rare genetic disease with an estimated prevalence of 2 cases per 100,000 people in the United States [1]. Cases of HAE with C1 esterase inhibitor (C1INH) deficiency (HAE-C1INH; HAE type I/II) arise from a mutation in the C1INH-encoding *SERPING1*, leading to either low C1INH levels or C1INH dysfunction [2, 3]. Cases of HAE with normal C1INH (HAE-nC1INH) involve a non-*SERPING1* mutation and normal C1INH levels and function [3]. Both HAE-C1INH and HAE-nC1INH disease subtypes are characterized by spontaneous, recurring attacks of subcutaneous and submucosal swelling that can occur anywhere in the body but most often affect the face, extremities, and gastrointestinal tract [2]. Involvement of the upper airways in an HAE attack is particularly high risk due to the potential for rapid airway obstruction [4]. HAE is associated with significant clinical, humanistic, and economic burdens, as HAE attacks can be fatal, can occur over multiple days, and often require management with specialist services, emergency department visits, or hospitalization [4, 5].

Therapy for HAE involves on-demand medications for the acute treatment of HAE attacks, short-term prophylaxis to be administered before exposure to a known HAE attack trigger, and long-term prophylaxis (LTP) for the overall reduction of HAE attack frequency and severity, improving disease control and health-related quality of life [6, 7]. On-demand treatment options include plasma-derived or recombinant C1INH, the plasma kallikrein inhibitor ecallantide and the bradykinin B2 receptor antagonist icatibant [7]. Until recently, targeted LTP therapies in the United States, including plasma-derived C1INH or the anti-kallikrein monoclonal antibody lanadelumab, required intravenous or subcutaneous administration [7].

In December 2020, the orally administered plasma kallikrein inhibitor berotralstat received US Food and Drug Administration (FDA) approval as an LTP therapy for the prevention of HAE attacks among patients with HAE aged ≥ 12 years [8]. In the berotralstat clinical program, comprising the APeX-1 [9], APeX-2 [10–12], APeX-J [13, 14], and APeX-S [15] trials, berotralstat reduced both the rate of HAE attacks and the use of on-demand medications while maintaining a favorable safety profile and improving patient quality of life [16]. Given the majority of patients with HAE prefer oral versus injectable administration, berotralstat has the potential to reduce treatment burden for patients with HAE on LTP [17–19].

Prior real-world studies have identified reduced HAE attack rates among patients with HAE following berotralstat initiation [20–28]. Nevertheless, the present study is the first to comprehensively evaluate attack rates following the initiation of berotralstat in patients with HAE of all types and across baseline attack frequencies in the real world. HAE attack rates before berotralstat initiation may serve as an indicator for underlying disease burden or activity. Thus, this study assessed HAE attack rates before and after berotralstat initiation among patients with HAE-C1INH and HAE-nC1INH in the United States, further stratified by baseline attack frequency.

## Methods

### Data source

This retrospective study analyzed Specialty Pharmacy data from Optime Care, Inc. (Berwyn, PA), the sole dispenser of berotralstat in the United States, covering the period from December 15, 2020 to January 8, 2024. The database contains comprehensive berotralstat dispensing records, demographic information, laboratory measurements for the classification of HAE type (i.e., C1INH levels/function and C4 levels), and patient-reported HAE attack information from questionnaires administered at berotralstat onboarding and each refill. The on-boarding questionnaire was administered to patients upon their initiation of berotralstat, with follow-up questionnaires delivered by phone or mobile application approximately every 28 days before the next berotralstat dispensing (approximately every 28 days, which corresponds to the usual days’ supply of each berotralstat package).

### Study design and population

This was a retrospective pre–post study where patients served as their own controls to eliminate confounding by factors that do not change over time [29]. The first berotralstat dispensing date was defined as the index date, and the baseline period was defined as the 90 days before the index date. The follow-up period varied for each patient and spanned from the index date until the last dispensing of berotralstat, up to a maximum of 18 months (540 days), and was segmented into 90-day intervals to facilitate comparison with the baseline period. Monthly HAE attack rates were compared between the 90-day follow-up intervals and the baseline period. All analyses were stratified by HAE type, with further analyses stratified by monthly baseline attack frequency (i.e.,≥ 5, 2–4, 1, and 0 attacks/month).

Patients included in the study had (i) ≥ 2 dispensings of berotralstat between December 15, 2020 and January 8, 2024 (end of data availability) and (ii) *International Classification of Diseases, Tenth Revision, Clinical Modification* (ICD-10-CM) diagnosis code D84.1 “Defects in the complement system.” Patients were excluded if they (a) were enrolled in a pre-launch clinical trial or expanded access program, (b) were < 12 years of age at index, (c) had <  90 days of follow-up, or (d) initiated berotralstat on Quick Start, did not transition to another fill type (i.e., the Patient Assistance Program [PAP] or a paid dispensing), and also had normal C1INH levels and function. To evaluate HAE attacks, patients were required to have a self-assessment of HAE attacks at both baseline and ≥ 1 follow-up interval; to be included in the analysis of a given 90-day follow-up interval, patients were required to have a self-assessment of HAE attacks during the given interval and a follow-up period extending through at least the last day of the same interval.

Patients were categorized into one of three HAE type subgroups based on laboratory measurements of C1INH levels, C1INH function, and C4 levels: patients with C1INH deficiency (HAE-C1INH; HAE type I/II), patients with normal C1INH (HAE-nC1INH), or undetermined HAE type. Patients with undetermined HAE type may have had missing laboratory values in the database or a combination of values that could not reliably be classified. Supplementary Table 1, Additional File 1 presents a detailed description of the HAE type classification.

Sensitivity analyses were conducted using a 30-day recall period at baseline and 30-day follow-up intervals to assess the robustness of attack rate reduction findings using a shorter baseline recall period.

### Study outcomes

Patient demographics were reported at index, including age, sex, region of residence, and median household income based on patient ZIP code. Clinical characteristics were reported at index, including patient weight, prescribing healthcare practitioner specialty, dosage of initial berotralstat dispensing (110 or 150 mg), and whether patients reported LTP experience prior to berotralstat initiation.

Monthly HAE attack rates, based on patient-reported HAE attacks, were assessed both in the 90-day baseline period and across the 90-day follow-up intervals. Non-numeric HAE attack self-assessments (e.g., “10 or more”) were imputed based on the mean of corresponding numeric responses. The maximum rate of HAE attacks that a patient could experience was assumed to be 1 attack every 2 days (e.g., 15 total attacks over 30 days), with attack rates over this maximum trimmed. At baseline, patients were asked how many attacks they had experienced over two time periods: the past 90 days and the past 30 days. The monthly baseline attack rate was calculated for a given patient by dividing the number of attacks they reported in the 90 days before berotralstat initiation by 3; in the rare instances when the value for 90 days was missing, the value for 30 days was used. Monthly baseline attack frequency categories were defined as follows:  ≥ 5 (i.e.,  ≥4.5 attacks/month), 2–4 (i.e., ≥ 1.5 to <4.5 attacks/month), 1 (i.e.,  ≥ 0.5 to <1.5 attacks/month), and 0 (i.e., <0.5 attacks/month).

Attack rates during follow-up were calculated as the number of HAE attacks reported by the patient (i.e., numerator) over the recall period (i.e., denominator), defined by the minimum of either (a) the time from the previous berotralstat shipment date, or (b) 30 days, reflecting the questionnaire format. Attacks were assumed to have occurred uniformly over the recall period (denominator) and were allocated across two fixed 90-day intervals proportionally to the extent of overlap of the recall period over the two intervals. The total number of attacks in each interval was divided by three to obtain the monthly attack rate.

Additionally, as patients had variable-length follow-up periods, where not all patients had  ≥ 18 months of follow-up, the reasons for sample size decreases across each 90-day interval were reported. Potential reasons included (a) patients reached the end of data availability without evidence of discontinuation (i.e., the proximity between the last day of supply and the end of data availability did not allow for the determination of whether patients continued to use berotralstat), (b) patients discontinued berotralstat before the end of the next interval (defined as a gap of  ≥ 60 days between the end of supply of a dispensing and the ship date of the next dispensing, or between the end of supply of the last dispensing and the end of data availability), (c) patients had a berotralstat dispensing but no self-assessment of attacks during the next follow-up interval, and (d) patients discontinued berotralstat before the end of the next interval but re-initiated in a subsequent interval. The proportions of patients in each interval who were not included in the subsequent interval due to these reasons were reported.

### Statistical analysis

Baseline demographics and clinical characteristics were described using mean, standard deviation (SD), and median values for continuous variables and frequencies and proportions for categorical variables.

Mean and median HAE attack rates were reported per-patient-per-month at baseline and for each 90-day interval up to 18 months of follow-up (540 days). Monthly attack rates were compared between the 90-day follow-up periods and the 90-day baseline period using mean differences (MDs), 95% confidence intervals (CIs), and *P*-values derived from generalized estimating equations (GEE) linear regression models with robust standard errors. The same models were used to analyze patients with ≥ 5, 2–4, or 1 baseline attack/month. For patients with 0 baseline attacks/month, the proportion of patients with 0 follow-up attacks at each interval was reported.

## Results

### Demographics and clinical characteristics

The study population consisted of 390 patients with HAE-C1INH and 311 with HAE-nC1INH (Supplementary Fig. 1, Additional File 1). For patients with HAE-C1INH, 70 (17.9%), 110 (28.2%), 102 (26.2%), and 108 (27.7%) patients had  ≥ 5, 2–4, 1, or 0 baseline attacks/month, respectively. For patients with HAE-nC1INH, 134 (43.1%), 76 (24.4%), 67 (21.5%), and 34 (10.9%) patients had  ≥ 5, 2–4, 1, or 0 baseline attacks/month, respectively.

The mean age of patients with HAE-C1INH was 39.3 years and 64.1% of patients were female, while the mean age of patients with HAE-nC1INH was 48.1 years and 77.5% were female (Table 1). Mean age across baseline attack frequency categories for patients with HAE-C1INH ranged from 37.2 to 42.5 years, while for patients with HAE-nC1INH, mean age ranged from 46.4 to 53.2 years (Supplementary Table 2, Additional File 1). The most common region of residence at index was the South (41.0–56.6%), corresponding to the most populous region in the United States [30]. In total, 95.9% and 91.0% of patients with either HAE-C1INH or HAE-nC1INH, respectively, were initiated on berotralstat 150 mg. The majority of physicians who first prescribed berotralstat were allergists or immunologists, comprising 94.4% of patients with HAE-C1INH and 89.4% of patients with HAE-nC1INH. More than one half of patients with HAE were LTP-naïve at index, both overall (HAE-C1INH: 54.1%, HAE-nC1INH: 68.2%) and across all baseline attack frequencies, except for patients with HAE-C1INH and 0 baseline attacks/month (30.6%).

**Table 1:** Patient demographics and clinical characteristics

Patient characteristics	Patients with HAE-C1INH^a^	Patients with HAE-nC1INH^a^
	(N = 390)	(N = 311)
*Demographics*		
Age at index, years, mean ± SD [median]	39.3 ± 19.0 [37]	48.1 ± 16.8 [49]
*Age category at index, years, n (%)*		
12–17	58 (14.9)	12 (3.9)
18–24	59 (15.1)	24 (7.7)
25–29	26 (6.7)	10 (3.2)
30–34	35 (9.0)	28 (9.0)
35–39	34 (8.7)	17 (5.5)
40–44	27 (6.9)	26 (8.4)
45–49	26 (6.7)	44 (14.1)
50–54	28 (7.2)	35 (11.3)
55–59	26 (6.7)	35 (11.3)
60–64	18 (4.6)	31 (10.0)
≥ 65	53 (13.6)	49 (15.8)
Female, n (%)	250 (64.1)	241 (77.5)
Median household income,^b^ 2022 USD, mean ± SD [median]	73,412 ± 25,904 [69,448]	76,893 ± 29,298 [68,566]
*Characteristics at index*		
Patient weight, kg., mean ± SD [median]	82 ± 23 [77]	84 ± 22 [82]
*Dosage of berotralstat at index date, n (%)*		
150 mg	374 (95.9)	283 (91.0)
110 mg	16 (4.1)	28 (9.0)
*LTP experience at index date,*^*c*^* n (%)*		
LTP experienced	179 (45.9)	99 (31.8)
LTP naïve	211 (54.1)	212 (68.2)
*Region,*^*d*^* n (%)*		
South	182 (46.7)	147 (47.3)
Midwest	91 (23.3)	76 (24.4)
Northeast	50 (12.8)	37 (11.9)
West	65 (16.7)	45 (14.5)
Puerto Rico	2 (0.5)	6 (1.9)
*HCP specialty, n (%)*		
Allergy/Immunology	368 (94.4)	278 (89.4)
Nurse practitioner	10 (2.6)	13 (4.2)
Other^e^	12 (3.1)	20 (6.4)

HAE, hereditary angioedema; HAE-C1INH, hereditary angioedema with C1 esterase inhibitor deficiency; HAE-nC1INH, hereditary angioedema with normal C1 esterase inhibitor; HCP, healthcare practitioner; LTP, long-term prophylaxis; PAP, patient assistance program; SD, standard deviation; USD, United States Dollar

^a^See Supplementary Table 1, Additional File 1 for the definition of HAE types

^b^Refers to the median income of households within the ZIP code of a patient’s place of residence. Income was inflation-adjusted to 2022 USD using the medical care component of the Consumer Price Index

^c^Patients were classified as LTP-experienced if they reported  ≥ 1 non-berotralstat LTP medication prior to index. Patients were classified as LTP-naïve if they did not report any non-berotralstat LTP medication prior to index

^d^All regions and divisions based on US Census Bureau designations

^e^Other HCP specialties included physician assistant, internal medicine, family practice, pediatrics, and rheumatology

### HAE attack rates among patients with HAE-C1INH

**Fig. 1:**
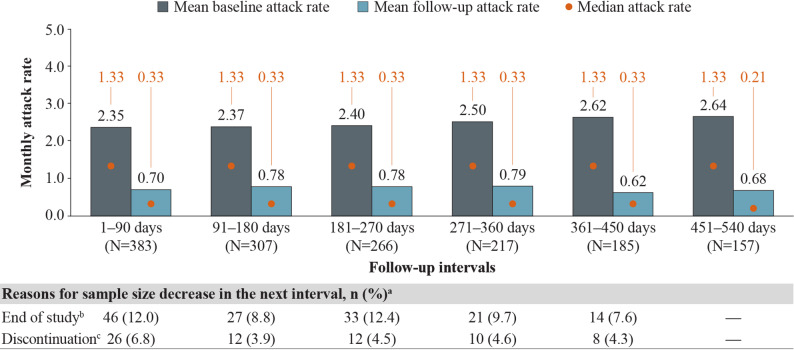
Monthly attack rates before and after berotralstat initiation. Measured across 90-day intervals among patients with HAE-C1INH. HAE, hereditary angioedema; HAE-C1INH, hereditary angioedema with C1 esterase inhibitor deficiency. ^a^ Other reasons for sample size decrease were no HAE attack report associated with dispensing in interval (0.8–3.2%) and discontinuation and later re-initiation (0.5–1.9%). ^b^ Patient reached end of study period (January 8, 2024) without evidence of discontinuation. ^c^ Discontinuation was defined as a gap of  ≥ 60 days between the end of supply of a dispensing and the ship date of the next dispensing, or between the last dispensing and the end of data availability (January 8, 2024)

**Fig. 2:**
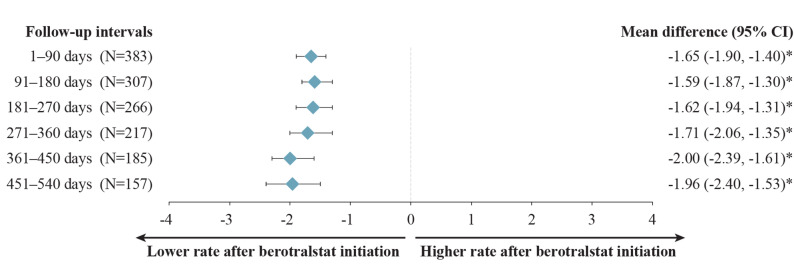
Mean differences in attack rates after versus before berotralstat initiation. Measured across 90-day intervals among patients with HAE-C1INH. CI, confidence interval; HAE, hereditary angioedema; HAE-C1INH, hereditary angioedema with C1 esterase inhibitor deficiency. *P < 0.05

Monthly HAE attack rates among patients with HAE-C1INH decreased significantly after berotralstat initiation and were sustained across every follow-up interval (Fig. 1). Mean (median) attack rates decreased from 2.50 (1.33) attacks/month at baseline to 0.79 (0.33) attacks/month at 12 months (271–360 days) of follow-up and from 2.64 (1.33) attacks/month at baseline to 0.68 (0.21) attacks/month at 18 months (451–540 days) of follow-up (Fig. 1), corresponding to statistically significant reductions (MD [95% CI]) of − 1.71 (−2.06, −1.35) attacks/month and − 1.96 (− 2.40,  −1.53) attacks/month, respectively (both *P* <  0.001) (Fig. 2). The primary reasons for sample size decreases among patients with HAE-C1INH were reaching the end of data availability (7.6–12.4% of patients within each 90-day interval) and treatment discontinuation (3.9–6.8% of patients within each 90-day interval) (Fig. 1).

**Fig. 3:**
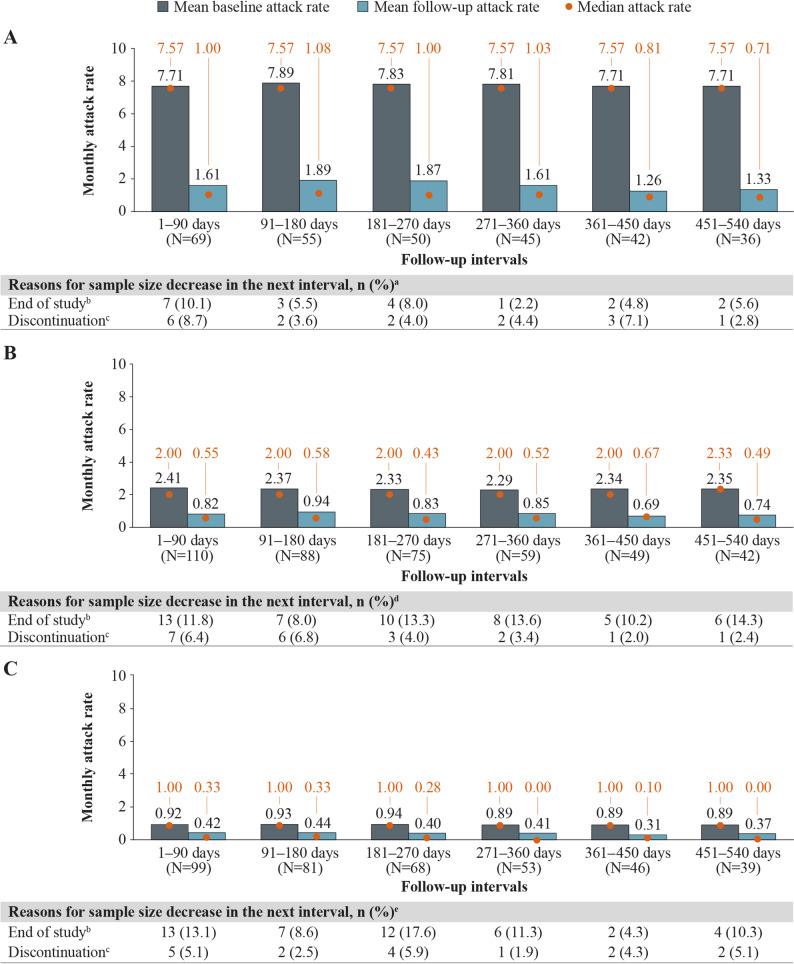
Monthly attack rates before and after berotralstat initiation, by monthly baseline attack frequency. Measured across 90-day intervals among patients with HAE-C1INH and (**A**)  ≥ 5 baseline attacks/month, **B** 2–4 baseline attacks/month, or **C** 1 baseline attack/month. Abbreviations: HAE, hereditary angioedema; HAE-C1INH, hereditary angioedema with C1 esterase inhibitor deficiency. ^a^ Sample size also decreased due to no self-assessment of HAE attacks linked with dispensing (0.0–2.8%). ^b^ Patient reached end of study period (January 8, 2024) without evidence of discontinuation. ^c^ Discontinuation was defined as a gap of  ≥ 60 days between the end of supply of a dispensing and the ship date of the next dispensing, or between the last dispensing and the end of data availability (January 8, 2024). ^d^ Sample size also decreased due to no self-assessment of HAE attacks linked with dispensing (0.0–2.4%) and discontinuation and later reinitiation (0.0–5.3%). ^e^ Sample size also decreased due to no self-assessment of HAE attacks linked with dispensing (0.0–6.5%) and discontinuation and later reinitiation (0.0–2.5%)

**Fig. 4:**
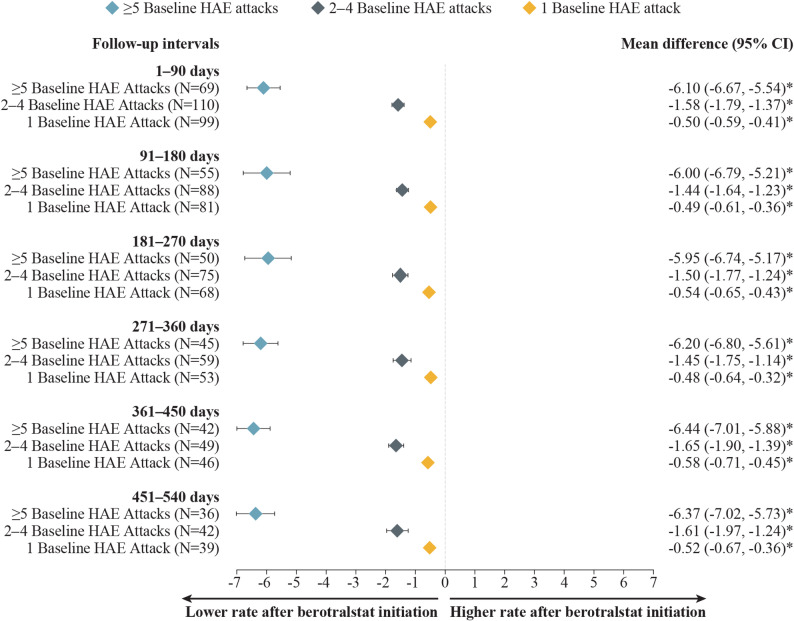
Mean differences in attack rates after versus before berotralstat initiation, by monthly baseline attack frequency. Measured across 90-day intervales among patients with HAE-C1INH and ≥ 5, 2–4, or 1 baseline attack/month. CI, confidence interval; HAE, hereditary angioedema; HAE-C1INH, hereditary angioedema with C1 esterase inhibitor deficiency. *P <  0.05

Patients with HAE-C1INH experiencing attacks at baseline had statistically significant mean attack rate reductions, regardless of baseline attack rate, with the largest improvement observed among patients with  ≥ 5 baseline attacks/month. For patients with HAE-C1INH and ≥ 5, 2–4, or 1 baseline attack/month, mean (median) attack rates decreased from 7.81 (7.57), 2.29 (2.00), and 0.89 (1.00) attacks/month at baseline, respectively, to 1.61 (1.03), 0.85 (0.52), and 0.41 (0.00) attacks/month at 12 months (271–360 days) of follow-up (Fig. 3). These decreases in HAE attack rates corresponded to statistically significant reductions (MD [95% CI]) of − 6.20 ( − 6.80, − 5.61),  − 1.45 ( − 1.75, − 1.14), and −0.48 (−0.64, −0.32) attacks/month among patients with  ≥ 5, 2–4, or 1 baseline attack/month, respectively (all *P* < 0.001) (Fig. 4). Patients with 0 baseline attacks/month maintained a similarly low level of attacks, which was sustained across follow-up intervals. At 12 months (271–360 days) and 18 months (451–540 days) of follow-up, 70% and 85% of patients, respectively, maintained an attack rate of 0 attacks/month (Supplementary Fig. 2, Additional File 1).

### HAE attack rates among patients with HAE-nC1INH

**Fig. 5:**
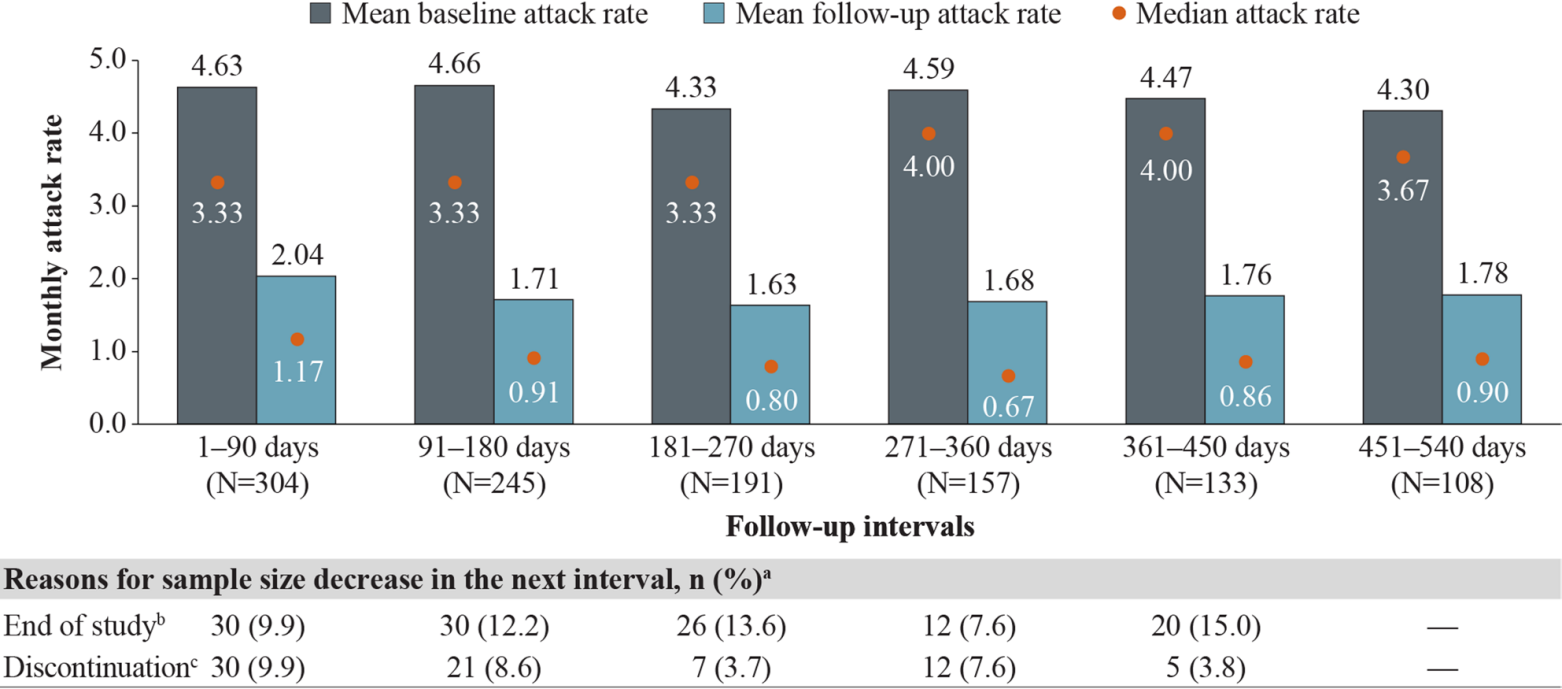
Monthly attack rates before and after berotralstat initiation. Measured across 90-day intervals among patients with HAE-nC1INH. HAE, hereditary angioedema; HAE-nC1INH, hereditary angioedema with normal C1 esterase inhibitor. ^a^ Other reasons for sample size decrease were no HAE attack report associated with dispensing in interval (0.0–1.0%) and discontinuation and later re-initiation (0.0–2.0%). ^b^ Patient reached end of study period (January 8, 2024) without evidence of discontinuation. ^c^ Discontinuation was defined as a gap of  ≥ 60 days between the end of supply of a dispensing and the ship date of the next dispensing, or between the last dispensing and the end of data availability (January 8, 2024)

**Fig. 6:**
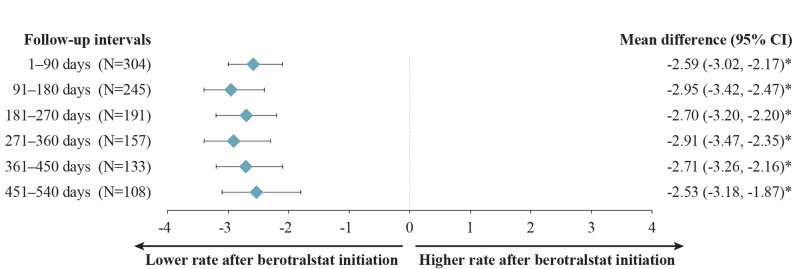
Mean differences in attack rates after versus before berotralstat initiation. Measured across 90-day intervals among patients with HAE-nC1INH. CI, confidence interval; HAE, hereditary angioedema; HAE-nC1INH, hereditary angioedema with normal C1 esterase inhibitor. *P < 0.05

Patients with HAE-nC1INH experienced statistically significant attack rate reductions, which were sustained across all follow-up intervals. Mean (median) attack rates decreased from 4.59 (4.00) attacks/month at baseline to 1.68 (0.67) attacks/month at 12 months (271–360 days) of follow-up and from 4.30 (3.67) attacks/month at baseline to 1.78 (0.90) attacks/month at 18 months (451–540 days) of follow-up (Fig. 5), corresponding to statistically significant reductions (MD [95% CI]) of − 2.91 (−3.47, −2.35) attacks/month and −2.53 ( −3.18, −1.87) attacks/month, respectively (both *P* <  0.001) (Fig. 6). The primary reasons for sample size decreases among patients with HAE-nC1INH were reaching the end of data availability (9.9–15.0% of patients within each 90-day interval) and treatment discontinuation (3.7–9.9% of patients within each 90-day interval).

**Fig. 7:**
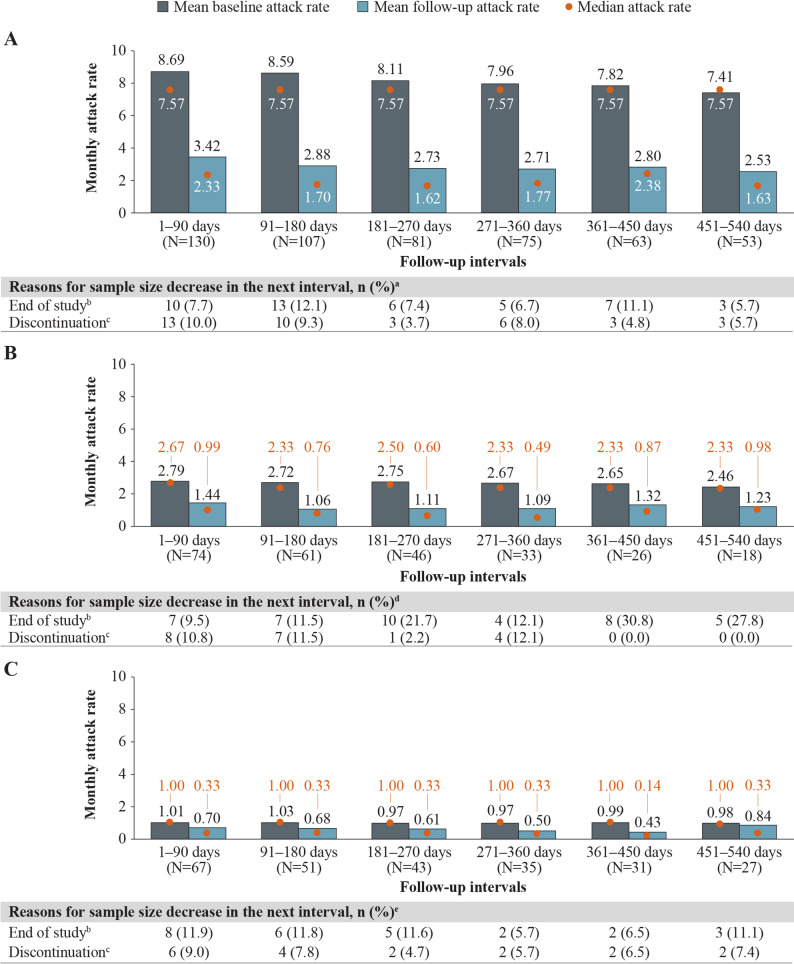
Monthly attack rates before and after berotralstat initiation, by monthly baseline attack frequency. Measured across 90-day intervals among patients with HAE-nC1INH and (**A**)  ≥ 5 baseline attack/month (**B**) 2–4 baseline attacks/month, or (**C**) 1 baseline attacks/month. HAE, hereditary angioedema; HAE-nC1INH, hereditary angioedema with normal C1 esterase inhibitor. ^a^ Other reasons for sample size decrease were no self-assessment of HAE attacks associated with dispensing in interval (0.0–1.9%) and discontinuation with later reinitiation (0.0–3.7%). ^b^ Patient reached end of study period (January 8, 2024) without evidence of discontinuation. ^c^ Discontinuation was defined as a gap of  ≥ 60 days between the end of supply of a dispensing and the ship date of the next dispensing, or between the last dispensing and the end of data availability (January 8, 2024). ^d^ Other reasons for sample size decrease were no self-assessment of HAE attacks associated with dispensing in interval (0.0–5.6%) and discontinuation with later reinitiation (0.0–2.2%). ^e^ Other reasons for sample size decrease were no self-assessment of HAE attacks associated with dispensing in interval (0.0–3.7%) and discontinuation with later reinitiation (0.0–3.7%)

**Fig. 8:**
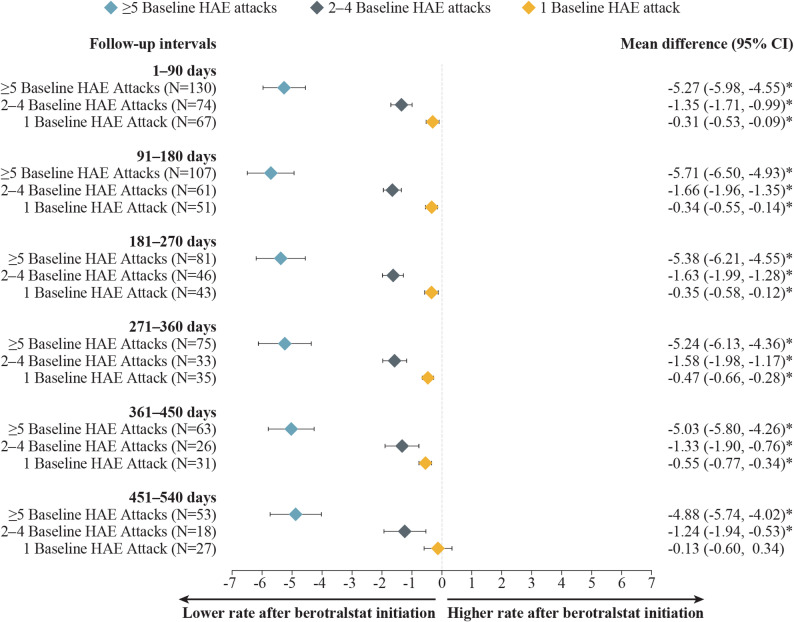
Mean differences in attack rates after versus before berotralstat initiation, by monthly baseline attack frequency. Measured across 90-day intervals among patients with HAE-nC1INH and ≥ 5, 2–4, or 1 baseline attack/month. CI, confidence interval; HAE-nC1INH, hereditary angioedema with normal C1 esterase inhibitor. *P <  0.05

Among patients with HAE-nC1INH, significant reductions were observed in all patients experiencing HAE attacks, regardless of baseline attack rate, with the largest improvements observed among patients with the highest baseline attack frequencies. For patients with HAE-nC1INH and ≥ 5, 2–4, and 1 baseline attack/month, mean (median) attack rates decreased from 7.96 (7.57), 2.67 (2.33), and 0.97 (1.00) attacks/month at baseline, respectively, to 2.71 (1.77), 1.09 (0.49), and 0.50 (0.33) attacks/month at 12 months (271–360 days) of follow-up (Fig. 7). These decreases in HAE attack rates corresponded to statistically significant reductions (MD [95% CI]) of −5.24 (−6.13, −4.36), −1.58 (−1.98, −1.17), and −0.47 ( −0.66,  −0.28) attacks/month among patients with  ≥ 5, 2–4, and 1 baseline attack/month, respectively (all *P* < 0.001) (Fig. 8). Patients with 0 baseline attacks/month maintained a similarly low level of attacks throughout follow-up. At 12 months (271–360 days) and 18 months (451–540 days) of follow-up, 71% and 70% of patients, respectively, maintained an attack rate of 0 attacks/month (Supplementary Fig. 3, Additional File 1).

### Sensitivity analysis using a 30-day baseline recall period

Using a 30-day baseline recall period among patients with HAE-C1INH, sustained attack rate reductions occurred across all follow-up intervals (Supplementary Fig. 4, Additional File 1). Mean (median) attack rates decreased from 3.15 (2.00) attacks/month at baseline to 0.87 (0.27) attacks/month at 12 months (271–360 days) of follow-up and 3.08 (2.00) attacks/month at baseline to 0.81 (0.00) attacks/month at 18 months (451–540 days) of follow-up (Supplementary Fig. 4, Additional File 1), corresponding to statistically significant reductions (MD [95% CI]) of − 2.28 ( − 2.76,  −1.80) attacks/month and −2.27 (−2.84,  −1.69) attacks/month, respectively (both *P* < 0.001) (Supplementary Fig. 5, Additional File 1). Patients with HAE-nC1INH also experienced sustained attack rate reductions across follow-up intervals (Supplementary Fig. 6, Additional File 1). Mean (median) attack rates decreased from 5.43 (4.00) attacks/month at baseline to 1.68 (0.67) attacks/month at 12 months (271–360 days) of follow-up and 5.26 (3.00) attacks/month at baseline to 2.00 (0.70) attacks/month at 18 months (451–540 days) of follow-up (Supplementary Fig. 6, Additional File 1), corresponding to statistically significant reductions (MD [95% CI]) of −3.75 ( −4.43,  −3.06) attacks/month and −3.26 (−4.21, −2.32) attacks/month, respectively (both *P* < 0.001) (Supplementary Fig. 7, Additional File 1).

## Discussion

In this real-world study, patients with HAE had significantly lower rates of attacks following berotralstat initiation regardless of HAE type (HAE-C1INH or HAE-nC1INH) or baseline attack rate. Further, HAE attack rates decreased significantly in the first 3 months after berotralstat initiation and remained significantly lower compared with baseline throughout the 18-month follow-up period. When stratified by attack rate prior to berotralstat initiation, patients with  ≥ 1 attack/month at baseline experienced significant and sustained reductions in attack rates after berotralstat initiation, regardless of baseline attack rate or HAE type. The majority of patients with 0 baseline attacks/month maintained an attack rate of 0 attacks/month following berotralstat initiation.

This study found that HAE attack rates among patients with HAE-C1INH were reduced from an average of 1.3 attacks/month in the baseline period to 0.3 attacks/month during days 1–90 of berotralstat treatment, with HAE attack rate reductions maintained throughout all 90-day follow-up intervals to 540 days (18 months). Prior analyses using sole-source pharmacy data through June 2023 that were presented at scientific conferences have also shown sustained reductions to HAE attack rates following berotralstat initiation among patients with HAE-C1INH in the United States [23–27]. Among other real-world studies conducted outside of the United States, berotralstat initiation has also been consistently associated with reduced HAE attack rates over the first 6 months of treatment among patients with HAE-C1INH. The present study expands on these analyses by extending data availability to January 2024; conducting rigorous statistical testing to assess the significance of attack rate decreases; and stratifying patients by HAE type—defined by laboratory measures (C1INH levels, C1INH function, and C4 levels)—along with monthly baseline attack frequency. Additionally, the use of sole-source Optime Care Specialty Pharmacy data allowed for the analysis of a large patient sample despite the rarity of HAE.

Importantly, the significant and sustained reductions to HAE attack rates among patients with HAE-C1INH following berotralstat initiation in this real-world study expand on those observed in the randomized, double-blind, placebo-controlled, phase III APeX-2 trial, wherein patients with HAE-C1INH and a pre-berotralstat attack rate of  ≥ 1 HAE attack/month (i.e.,  ≥ 2 attacks in the prior 56 days) received berotralstat 110 mg, berotralstat 150 mg, or placebo for 24 weeks [10]. The APeX-2 trial Part 2 followed patients for an additional 24 weeks, with patients in the placebo group re-randomized to receive either berotralstat 110 or 150 mg, while patients initially randomized to berotralstat continued the same treatment dose [11]. Among those who received berotralstat 150 mg for the entire 48-week period, mean HAE attack rates declined by 1.36 attacks/month at week 24 and by 2.00 attacks/month at week 48 [11]. The current study identified similar outcomes in a real-world setting despite including patients with a baseline HAE attack rate of 0 attacks/month—patients with HAE-C1INH achieved an average reduction of 1.71 attacks/month after 12 months of berotralstat treatment, irrespective of prior LTP. These results also expand on previous studies of berotralstat efficacy and effectiveness among participants with HAE-C1INH, including APeX-J, APeX-S, and the APeX-2 open-label extension, which demonstrated sustained reductions in HAE attack rates up to 26 months of berotralstat treatment [12–15].

The HAE-nC1INH disease subtype is understudied in clinical trials and in the real world. In a previous retrospective case series among patients with HAE-nC1INH in the United States, 13 of 15 patients reported improved HAE attack frequency, severity, or both with berotralstat treatment [28]. Similarly, a 2-patient case series of HAE-nC1INH conducted in Germany and Austria determined that mean HAE attack rates declined from 3.8 attacks/month at baseline to 1 attack/month at 3 months of berotralstat treatment [20]. The present study population consisted of 311 patients with HAE-nC1INH and is therefore the most comprehensive analysis of HAE attack rates before and after LTP initiation among patients with this disease subtype. Baseline attack rates were high among patients with HAE-nC1INH. The significant reduction to HAE attack rates among patients with HAE-nC1INH identified in the present study supports the current use of berotralstat as LTP for all patients with HAE, irrespective of disease subtype. Further, significant reductions to HAE attack rates among patients with HAE-nC1INH suggest that most of these cases are bradykinin-mediated and respond to berotralstat, reinforcing the importance of clinicians considering HAE as a potential diagnosis, even when C1INH levels and function are normal.

This is the first real-world study to evaluate HAE attack rates following berotralstat initiation stratified by HAE attack rate at baseline, showing that berotralstat treatment improved HAE attack rates among all patients with ≥ 1 attack/month at baseline (among  ≥ 5, 2–4, and 1 attack/month subgroups) and maintained HAE attack rates among patients with 0 attacks/month at baseline. While the greatest reductions in HAE attack rates were observed among patients with the highest HAE attack rates prior to berotralstat initiation, all patients who experienced baseline HAE attacks had significant reductions after berotralstat initiation. These results are generally consistent with those of the APeX-2 clinical trial, which also stratified by baseline attack rate [10].

HAE is a heterogeneous disease, and patients experience varying rates of attacks; however, LTP medication use before berotralstat initiation in the present study could also have influenced patients’ baseline attack rates. Specifically, patients with prior LTP experience, including those who directly switched from another LTP to berotralstat, would be anticipated to already be experiencing low HAE attack rates at baseline (0 or 1 attack/month). Indeed, patient-reported LTP experience any time before berotralstat initiation was prevalent among patients with 0 (HAE-C1INH: 69%; HAE-nC1INH: 38%) and 1 (HAE-C1INH: 43%; HAE-nC1INH: 27%) attack/month at baseline. The maintenance of low HAE attack rates with berotralstat treatment among patients with 0 baseline attacks/month and the reduction in HAE attack rate observed among patients with 1 baseline attacks/month in the present study suggest that switching to berotralstat from either intravenous or subcutaneous LTP therapy did not adversely impact disease control. Further research investigating the impact of berotralstat among patients who switched from another LTP is warranted.

The results of this study should be interpreted in light of some limitations. As there is a lack of standard diagnostic criteria for HAE-nC1INH [31], HAE type was determined according to C1INH levels, C1INH function, and C4 levels based on prior literature and clinical input (Supplementary Table 1, Additional File 1) [32]. The significant reductions in attack rates with berotralstat treatment among both the HAE-C1INH and HAE-nC1INH cohorts suggests that most cases were bradykinin-mediated. Additionally, some patients could not be classified as either HAE-C1INH or HAE-nC1INH due to missing or contradicting laboratory values, potentially driven by data entry errors or omission, and were therefore not included in the analyses. Patients completed surveys until the last dispensing of berotralstat, so information on HAE attacks was not collected after treatment discontinuation, though sample size decrease across follow-up intervals was driven by end of data availability more than treatment discontinuation. Information on prior HAE medication use was available through patient self-report, though the precise timing of when the medications were used prior to berotralstat initiation was not reliably collected; reason for berotralstat initiation was not collected. Additionally, the record of berotralstat dispensing in Optime Care Specialty Pharmacy data did not guarantee that the medication was consumed or taken as prescribed, which is a limitation common to analyses of pharmacy dispensing data. To allocate attacks within fixed 90-day intervals, HAE attack rates were assumed to have occurred uniformly over the recall period, despite attacks often clustering around periods of acute stress; however, there was no way to determine when in the recall period such clusters may have occurred. For the imputation of non-numeric HAE attack values, responses (e.g., “10 or more”) were imputed based on the average of corresponding numeric responses; this may not have accurately reflected the experience of a given patient. With the goal of only including patients with HAE in the study population, patients with a primary diagnosis of angioneurotic edema (T78.3) instead of defects in the complement system (D84.1) were excluded, which may have inadvertently excluded some patients with HAE; however, this criterion excluded very few patients.

## Conclusions

This large real-world study found that berotralstat was associated with significant and sustained reductions in HAE attack rates. When further categorizing patients by baseline attack frequency, significant reductions were observed among patients who experienced baseline attacks, regardless of baseline attack rate, while patients with 0 baseline attacks/month maintained low HAE attack rates after initiating treatment with berotralstat.

## Supplementary Information

Below is the link to the electronic supplementary material.

Supplementary Material 1.

## Data Availability

The data that support the findings of this study are available from Optime Care, Inc. (Berwyn, PA). Restrictions apply to the availability of these data, which were used under license for this study.

## Abbreviations

C1INH: C1 esterase inhibitor
CI: Confidence interval
GEE: Generalized estimating equations
HAE: Hereditary angioedema
HAE-C1INH: Hereditary angioedema with C1 esterase inhibitor deficiency
HAE-nC1INH: Hereditary angioedema with normal C1 esterase inhibitor
ICD-10-CM: International classification of diseases, tenth revision, clinical modification
LTP: Long-term prophylaxis
MD: Mean difference
PAP: Patient assistance program
SD: Standard deviation
US FDA: United States food and drug administration
