# Supplementary material for: Attack rate reductions following berotralstat initiation among US patients with hereditary angioedema in the real-world
Source: Allergy Asthma Clin Immunol. 2026 Jan 25;22:10. doi: 10.1186/s13223-025-01005-x (PMC13007376; doi:10.1186/s13223-025-01005-x)
Supplement: Supplementary file 1 — Supplementary Material 1. [file 13223_2025_1005_MOESM1_ESM.docx]

# Additional File 1

## Supplementary Table 1. HAE type assignment using laboratory measurements

| **HAE type^a^** | **C1INH levels**^b^ | **C1INH function**^c^ | **C4 levels**^d^ |
| --- | --- | --- | --- |
| HAE-C1INH (type I) | Low | Low | Low, Normal, High, Missing |
| HAE-C1INH (type I) | Low | Equivocal | Low, Missing |
| HAE-C1INH (type I) | Low | Missing | Low |
| HAE-C1INH (type II) | Normal, High | Low | Low, Missing |
| HAE-C1INH (type II) | Normal, High | Equivocal | Low |
| HAE-C1INH (type I/II; cannot distinguish) | Missing | Low | Low, Missing |
| HAE-nC1INH | Normal, High | Normal | Low, Normal, High, Missing |
| HAE-nC1INH | Normal, High | Equivocal | Normal, High |
| HAE-nC1INH | Missing | Normal | Normal, High |
| Undetermined | Missing | Low | Normal, High |
| Undetermined | Missing | Normal | Low, Missing |
| Undetermined | Missing | Equivocal | Low, Normal, High, Missing |
| Undetermined | Low | Missing | Normal, High, Missing |
| Undetermined | Normal, High | Missing | Low, Normal, High, Missing |
| Undetermined | Missing | Missing | Low, Normal, High, Missing |
| Undetermined | Normal, High | Low | Normal, High |
| Undetermined | Low | Normal | Low, Normal, High, Missing |
| Undetermined | Low | Equivocal | Normal |

Abbreviations: HAE, hereditary angioedema; HAE-C1INH, hereditary angioedema with C1 esterase inhibitor deficiency; HAE-nC1INH, hereditary angioedema with normal C1 esterase inhibitor.

a. HAE type was assigned based on observed laboratory values for C1INH levels, C1INH function, and C4 levels. For patients with multiple laboratory measurements of a given biomarker, the latest measurement of the biomarker was used. Some patients had missing values for one or more biomarkers.

b. C1INH serum levels were classified as low (<20 mg/dL), normal (20–39 mg/dL), and high (>39 mg/dL).

c. C1INH function was classified as low (<41%), equivocal (41–67%), and normal (>67%).

d. C4 levels were classified as low (<13 mg/dL), normal (13–52 mg/dL), and high (>52 mg/dL).

## Supplementary Table 2. Patient demographics and clinical characteristics by HAE type and monthly baseline attack frequency

| **Patient characteristics** | **Patients with HAE-C1INH^a^** | | | | |  | | **Patients with HAE-nC1INH^a^** | | | | |  | |
| --- | --- | --- | --- | --- | --- | --- | --- | --- | --- | --- | --- | --- | --- | --- |
|  | **≥5 baseline attacks** | **2–4 baseline attacks** | **1 baseline attack** | **0 baseline attacks** |  | | **≥5 baseline attacks** | | **2–4 baseline attacks** | **1 baseline attack** | **0 baseline attacks** |  | |  |
|  | **(N=70)** | **(N=110)** | **(N=102)** | **(N=108)** |  | | **(N=134)** | | **(N=76)** | **(N=67)** | **(N=34)** |  | |  |
| **Demographics** |  |  |  |  |  | |  | |  |  |  |  | |  |
| Age at index, years, mean ± SD [median] | 40.1 ± 17.3 [36] | 37.2 ± 17.7 [36] | 37.8 ± 19.4 [36] | 42.5 ± 20.8 [41] |  | | 47.0 ± 15.3 [49] | | 46.4 ± 18.2 [48] | 49.7 ± 18.3 [50] | 53.2 ± 15.3 [59] |  | |  |
| Age category at index, years, n (%) |  |  |  |  |  | |  | |  |  |  |  | |  |
| 12–17 | 7 (10.0) | 18 (16.4) | 20 (19.6) | 13 (12.0) |  | | 3 (2.2) | | 4 (5.3) | 4 (6.0) | 1 (2.9) |  | |  |
| 18–24 | 6 (8.6) | 16 (14.5) | 18 (17.6) | 19 (17.6) |  | | 10 (7.5) | | 9 (11.8) | 5 (7.5) | 0 (0.0) |  | |  |
| 25–29 | 7 (10.0) | 10 (9.1) | 3 (2.9) | 6 (5.6) |  | | 5 (3.7) | | 2 (2.6) | 2 (3.0) | 1 (2.9) |  | |  |
| 30–34 | 12 (17.1) | 9 (8.2) | 7 (6.9) | 7 (6.5) |  | | 16 (11.9) | | 6 (7.9) | 4 (6.0) | 2 (5.9) |  | |  |
| 35–39 | 8 (11.4) | 11 (10.0) | 8 (7.8) | 7 (6.5) |  | | 7 (5.2) | | 3 (3.9) | 3 (4.5) | 4 (11.8) |  | |  |
| 40–44 | 6 (8.6) | 7 (6.4) | 7 (6.9) | 7 (6.5) |  | | 11 (8.2) | | 9 (11.8) | 4 (6.0) | 2 (5.9) |  | |  |
| 45–49 | 3 (4.3) | 7 (6.4) | 9 (8.8) | 7 (6.5) |  | | 19 (14.2) | | 11 (14.5) | 10 (14.9) | 4 (11.8) |  | |  |
| 50–54 | 4 (5.7) | 11 (10.0) | 6 (5.9) | 7 (6.5) |  | | 21 (15.7) | | 8 (10.5) | 4 (6.0) | 2 (5.9) |  | |  |
| 55–59 | 6 (8.6) | 7 (6.4) | 8 (7.8) | 5 (4.6) |  | | 14 (10.4) | | 8 (10.5) | 12 (17.9) | 1 (2.9) |  | |  |
| 60–64 | 3 (4.3) | 3 (2.7) | 4 (3.9) | 8 (7.4) |  | | 16 (11.9) | | 3 (3.9) | 5 (7.5) | 7 (20.6) |  | |  |
| ≥65 | 8 (11.4) | 11 (10.0) | 12 (11.8) | 22 (20.4) |  | | 12 (9.0) | | 13 (17.1) | 14 (20.9) | 10 (29.4) |  | |  |
| Female, n (%) | 54 (77.1) | 69 (62.7) | 66 (64.7) | 61 (56.5) |  | | 112 (83.6) | | 55 (72.4) | 48 (71.6) | 26 (76.5) |  | |  |
| Median household income,^b^ 2022 USD, mean ± SD [median] | 76,745 ± 27,773 [71,111] | 73,601 ± 25,562 [67,984] | 71,182 ± 22,647 [67,231] | 73,169 ± 27,927 [68,769] |  | | 76,882 ± 28,302 [67,201] | | 74,645 ± 28,636 [70,436] | 79,340 ± 29,786 [72,951] | 77,145 ± 34,258 [67,838] |  | |  |
| **Characteristics at index** |  |  |  |  |  | |  | |  |  |  |  | |  |
| Patient weight, kg., mean ± SD [median] | 83 ± 24 [77] | 81 ± 23 [80] | 78 ± 21 [76] | 85 ± 25 [79] |  | | 84 ± 23 [81] | | 84 ± 21 [84] | 82 ± 21 [81] | 85 ± 23 [82] |  | |  |
| Dosage of berotralstat at index date, n (%) |  |  |  |  |  | |  | |  |  |  |  | |  |
| 150 mg | 69 (98.6) | 104 (94.5) | 100 (98.0) | 101 (93.5) |  | | 118 (88.1) | | 70 (92.1) | 62 (92.5) | 33 (97.1) |  | |  |
| 110 mg | 1 (1.4) | 6 (5.5) | 2 (2.0) | 7 (6.5) |  | | 16 (11.9) | | 6 (7.9) | 5 (7.5) | 1 (2.9) |  | |  |
| LTP experience at index date,^c^ n (%) |  |  |  |  |  | |  | |  |  |  |  | |  |
| LTP experienced | 20 (28.6) | 40 (36.4) | 44 (43.1) | 75 (69.4) |  | | 44 (32.8) | | 24 (31.6) | 18 (26.9) | 13 (38.2) |  | |  |
| LTP naïve | 50 (71.4) | 70 (63.6) | 58 (56.9) | 33 (30.6) |  | | 90 (67.2) | | 52 (68.4) | 49 (73.1) | 21 (61.8) |  | |  |
| Region of residence,^d^ n (%) |  |  |  |  |  | |  | |  |  |  |  | |  |
| South | 35 (50.0) | 55 (50.0) | 46 (45.1) | 46 (42.6) |  | | 55 (41.0) | | 43 (56.6) | 35 (52.2) | 14 (41.2) |  | |  |
| Midwest | 13 (18.6) | 26 (23.6) | 27 (26.5) | 25 (23.1) |  | | 38 (28.4) | | 13 (17.1) | 14 (20.9) | 11 (32.4) |  | |  |
| Northeast | 10 (14.3) | 9 (8.2) | 16 (15.7) | 15 (13.9) |  | | 15 (11.2) | | 11 (14.5) | 7 (10.4) | 4 (11.8) |  | |  |
| West | 12 (17.1) | 19 (17.3) | 13 (12.7) | 21 (19.4) |  | | 24 (17.9) | | 7 (9.2) | 9 (13.4) | 5 (14.7) |  | |  |
| Puerto Rico | 0 (0.0) | 1 (0.9) | 0 (0.0) | 1 (0.9) |  | | 2 (1.5) | | 2 (2.6) | 2 (3.0) | 0 (0.0) |  | |  |
| HCP specialty, n (%) |  |  |  |  |  | |  | |  |  |  |  | |  |
| Allergy/Immunology | 66 (94.3) | 106 (96.4) | 98 (96.1) | 98 (90.7) |  | | 125 (93.3) | | 71 (93.4) | 55 (82.1) | 27 (79.4) |  | |  |
| Nurse practitioner | 2 (2.9) | 1 (0.9) | 2 (2.0) | 5 (4.6) |  | | 3 (2.2) | | 2 (2.6) | 6 (9.0) | 2 (5.9) |  | |  |
| Other^e^ | 2 (2.8) | 3 (2.7) | 2 (2.0) | 5 (4.7) |  | | 6 (4.4) | | 3 (3.9) | 6 (9.0) | 5 (14.7) |  | |  |

Abbreviations: HAE, hereditary angioedema; HAE-C1INH, hereditary angioedema with C1 esterase inhibitor deficiency; HAE-nC1INH, hereditary angioedema with normal C1 esterase inhibitor; HCP, healthcare practitioner; LTP, long-term prophylaxis; PAP, patient assistance program; SD, standard deviation; USD, United States Dollar.

a. See **Supplementary Table 1, Additional File 1** for the definition of HAE types.

b. Refers to the median income of households within the ZIP code of a patient's place of residence. Income was inflation-adjusted to 2022 USD using the medical care component of the Consumer Price Index.

c. Patients were classified as LTP-experienced if they reported ≥1 non-berotralstat LTP medication prior to index. Patients were classified as LTP-naïve if they did not report any non-berotralstat LTP medication prior to index.

d. All regions and divisions based on US Census Bureau designations.

e. Other HCP specialties included physician assistant, internal medicine, family practice, pediatrics, and rheumatology.

## Supplementary Figure 1. Patient disposition flowchart


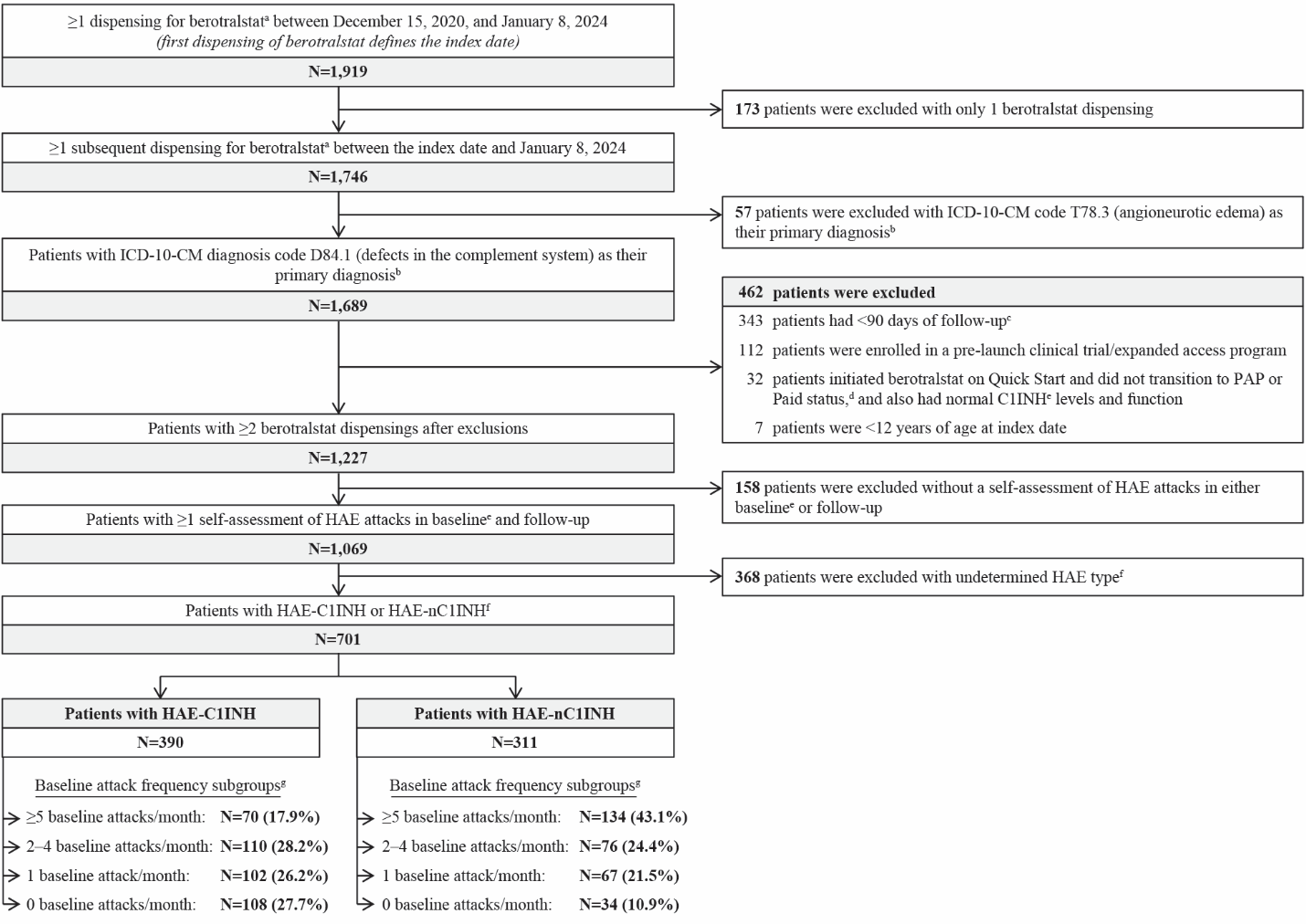


Abbreviations: C1INH, C1 esterase inhibitor; HAE, hereditary angioedema; HAE-C1INH, hereditary angioedema with C1 esterase inhibitor deficiency; HAE-nC1INH, hereditary angioedema with normal C1 esterase inhibitor; ICD-10-CM, International Classification of Diseases, Tenth Revision, Clinical Modification; NDC, National Drug Code; PAP, patient assistance program.

1. Berotralstat was identified using NDC codes 72769010201 (110 mg) and 72769010101 (150 mg)
2. Patients in the Optime Care Specialty Pharmacy data had a recorded primary diagnosis of D84.1 or T78.3.
3. The follow-up period spanned from the first to last berotralstat dispensing. Patient self-assessment data were not collected after the last berotralstat dispensing.
4. 100 patients initiated on Quick Start and did not transition to a PAP or Paid dispensing.
5. The 30-day baseline attack self-assessment was used when the 90-day baseline attack self-assessment was missing for 9 (2.3%) patients with HAE-C1INH and 4 (1.3%) patients with HAE-nC1INH.
6. See **Supplementary Table 1, Additional File 1** for the definition of HAE types. Normal C1INH serum levels were defined as measurements of 20–39 mg/dL, while normal C1INH function was defined as measurements >67%. Patients with undetermined HAE type may have had missing laboratory values in the database or a combination of laboratory values that could not reliably be classified.
7. Patients were included in the 0 baseline HAE attack category if they had <0.5 HAE attacks/month at baseline, 1 baseline HAE attack category if they had ≥0.5 to <1.5 HAE attacks/month at baseline, 2–4 baseline HAE attack category if they had ≥1.5 to <4.5 HAE attacks/month at baseline, and ≥5 baseline HAE attack category if they had ≥4.5 HAE attacks/month at baseline.

## Supplementary Figure 2. Proportion of patients with 0 follow-up attacks/month among patients with 0 baseline attacks/month and HAE-C1INH


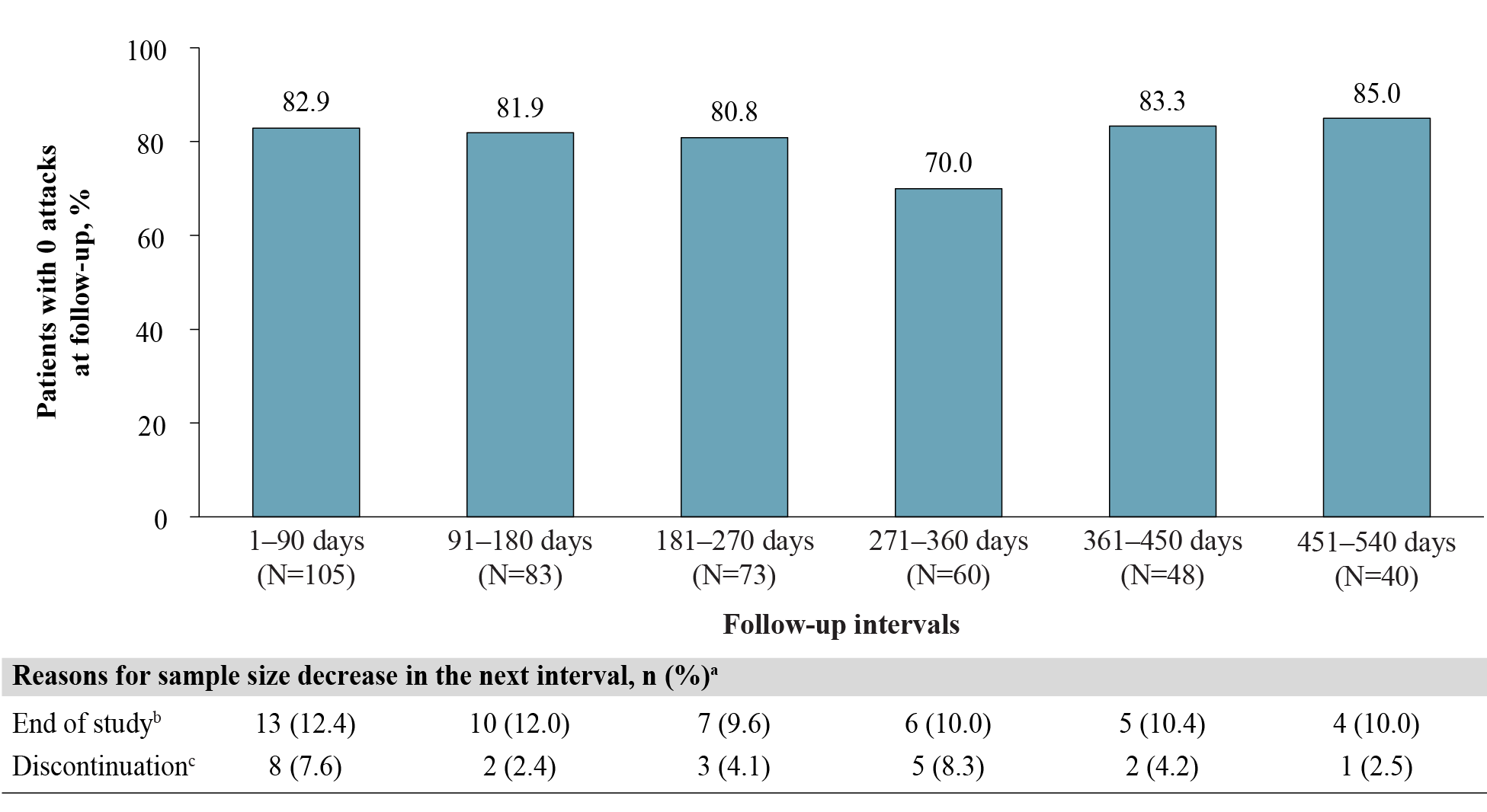


Abbreviations: HAE, hereditary angioedema; HAE-C1INH, hereditary angioedema with C1 esterase inhibitor deficiency.

a. Other reasons for sample size decrease were no self-assessment of HAE attacks associated with dispensing in interval (0.0%–3.3%), and discontinuation with later re-initiation (0.0%–2.1%).

b. Patient reached end of study period (January 8, 2024) without evidence of discontinuation.

c. Discontinuation was defined as a gap of ≥60 days between the end of supply of a dispensing and the ship date of the next dispensing, or between the last dispensing and the end of data availability (January 8, 2024).

## Supplementary Figure 3. Proportion of patients with 0 follow-up attacks/month among patients with 0 baseline attacks/month and HAE-nC1INH

**
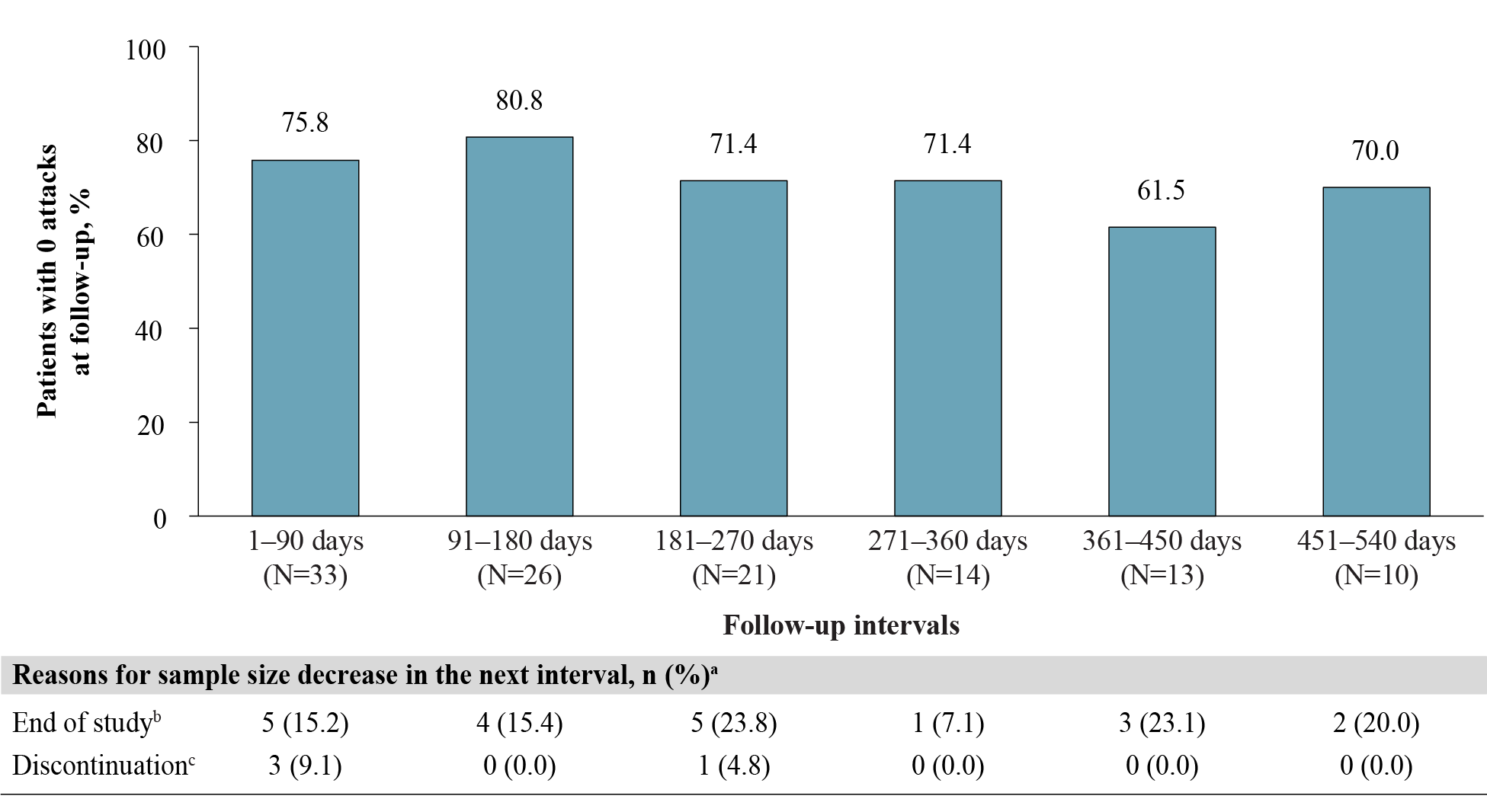
**

Abbreviation: HAE-nC1INH, hereditary angioedema with normal C1 esterase inhibitor.

a. Another reason for sample size decrease was no self-assessment of HAE attacks associated with dispensing in interval (0.0%–4.8%).

b. Patient reached end of study period (January 8, 2024) without evidence of discontinuation.

c. Discontinuation was defined as a gap of ≥60 days between the end of supply of a dispensing and the ship date of the next dispensing, or between the last dispensing and the end of data availability (January 8, 2024).

## Supplementary Figure 4. Monthly attack rates before and after berotralstat initiation across 30-day intervals among patients with HAE-C1INH


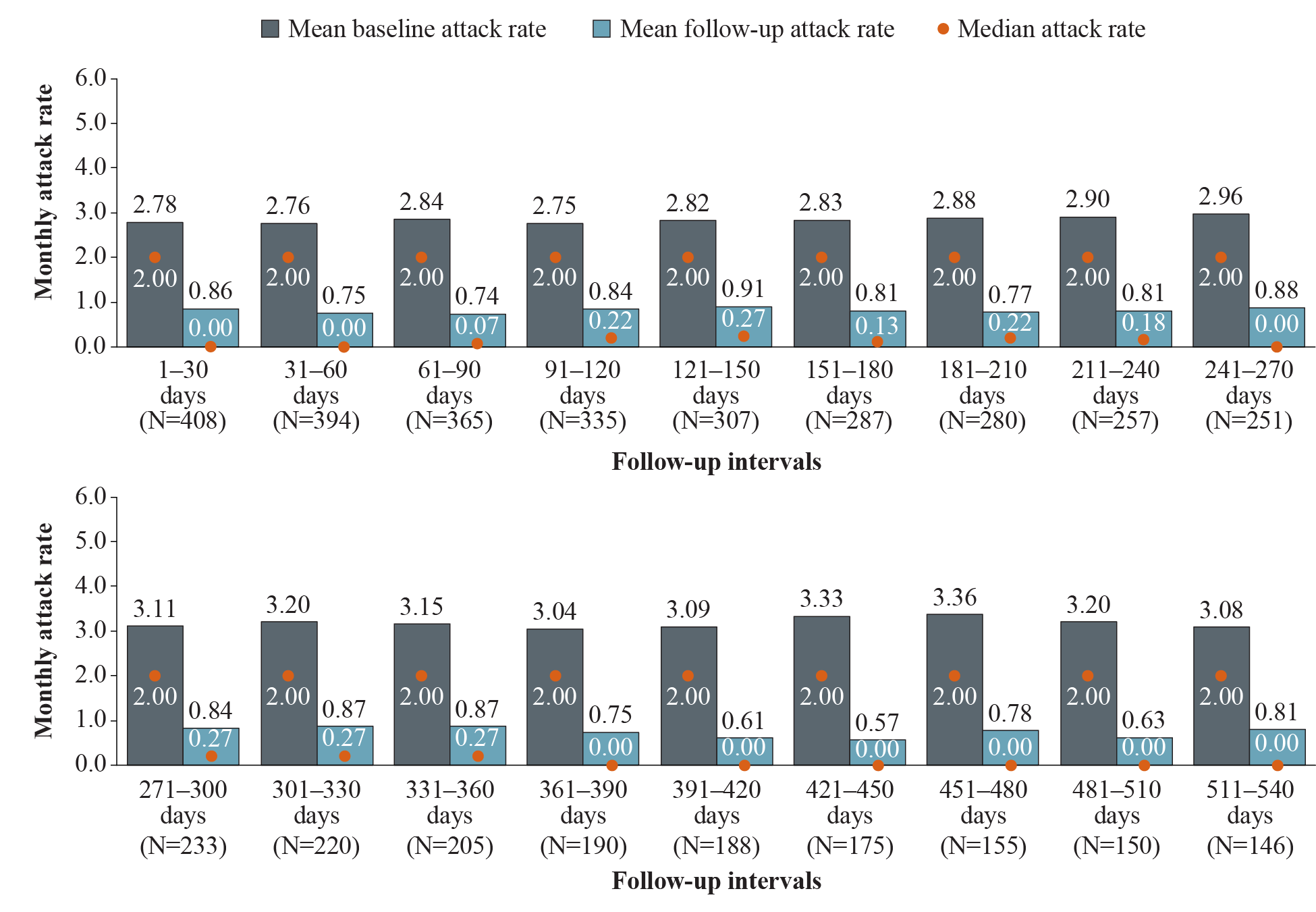


Abbreviations: HAE, hereditary angioedema; HAE-C1INH, hereditary angioedema with C1 esterase inhibitor deficiency.

## Supplementary Figure 5. Mean differences in attack rates after versus before berotralstat initiation across 30-day intervals among patients with HAE-C1INH


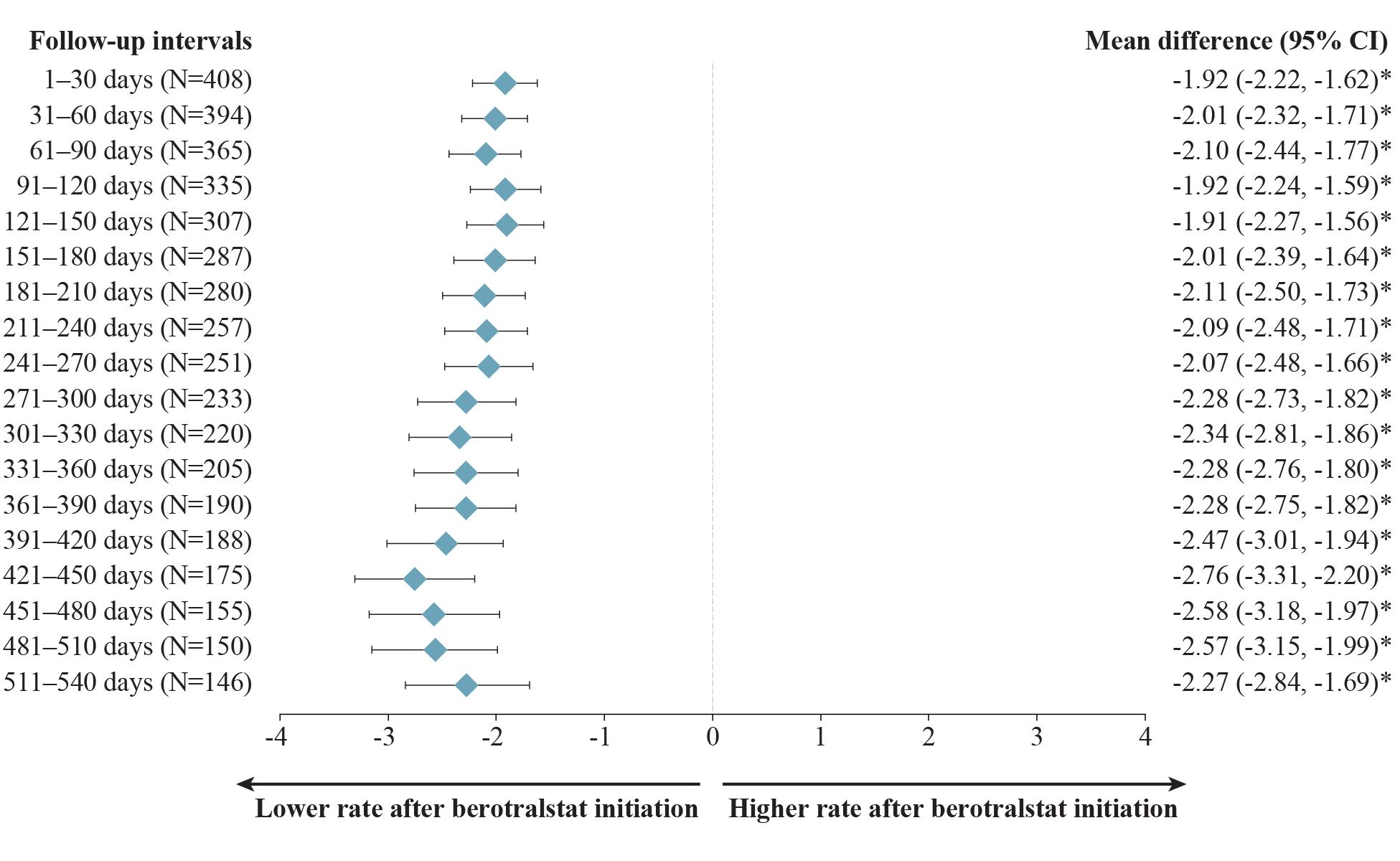


Abbreviations: CI, confidence interval; HAE, hereditary angioedema; HAE-C1INH, hereditary angioedema with C1 esterase inhibitor deficiency. **P*<.05.

## Supplementary Figure 6. Monthly attack rates before and after berotralstat initiation across 30-day intervals among patients with HAE-nC1INH


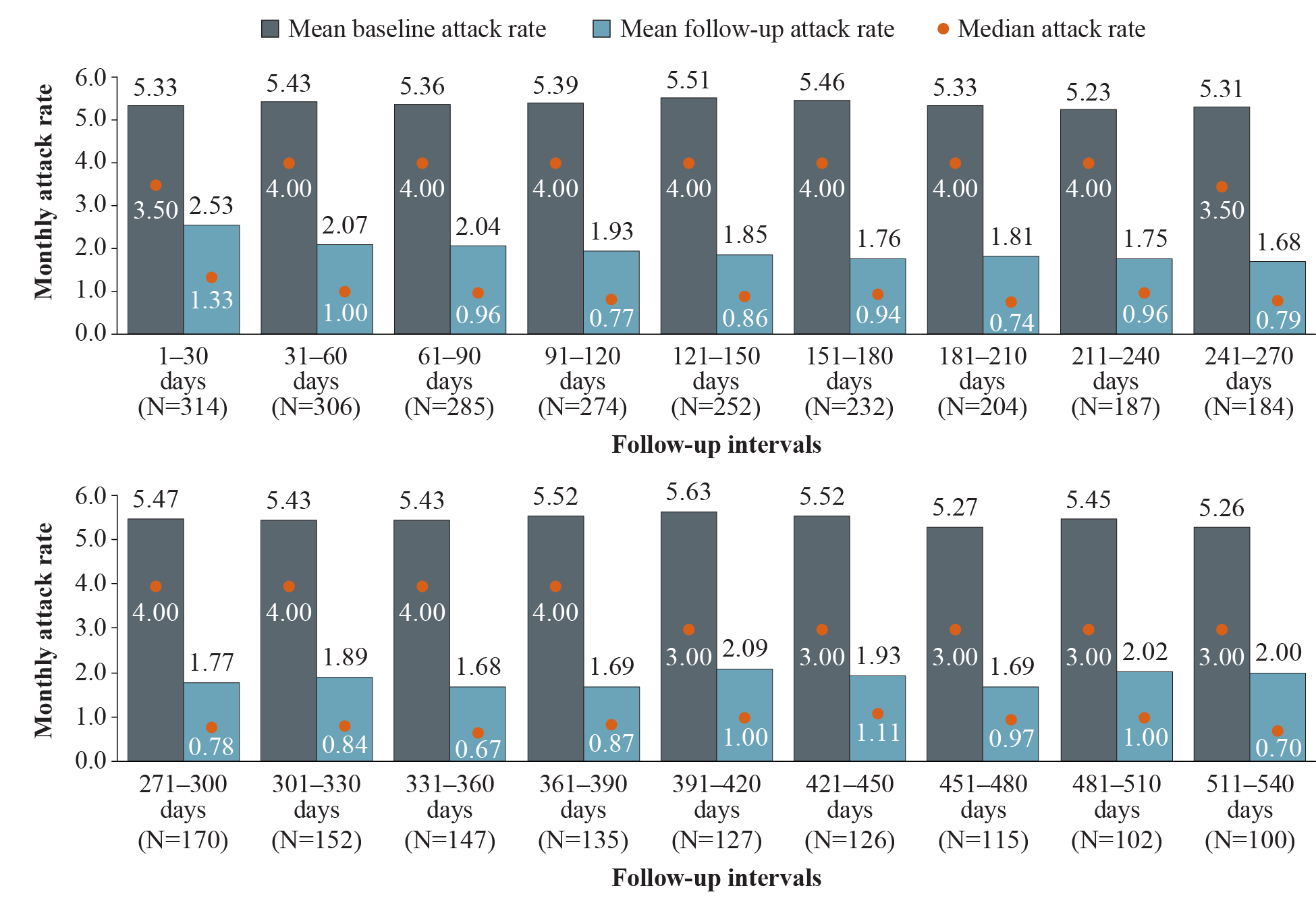


Abbreviation: HAE-nC1INH, hereditary angioedema with normal C1 esterase inhibitor.

## Supplementary Figure 7. Mean differences in attack rates after versus before berotralstat initiation across 30-day intervals among patients with HAE-nC1INH


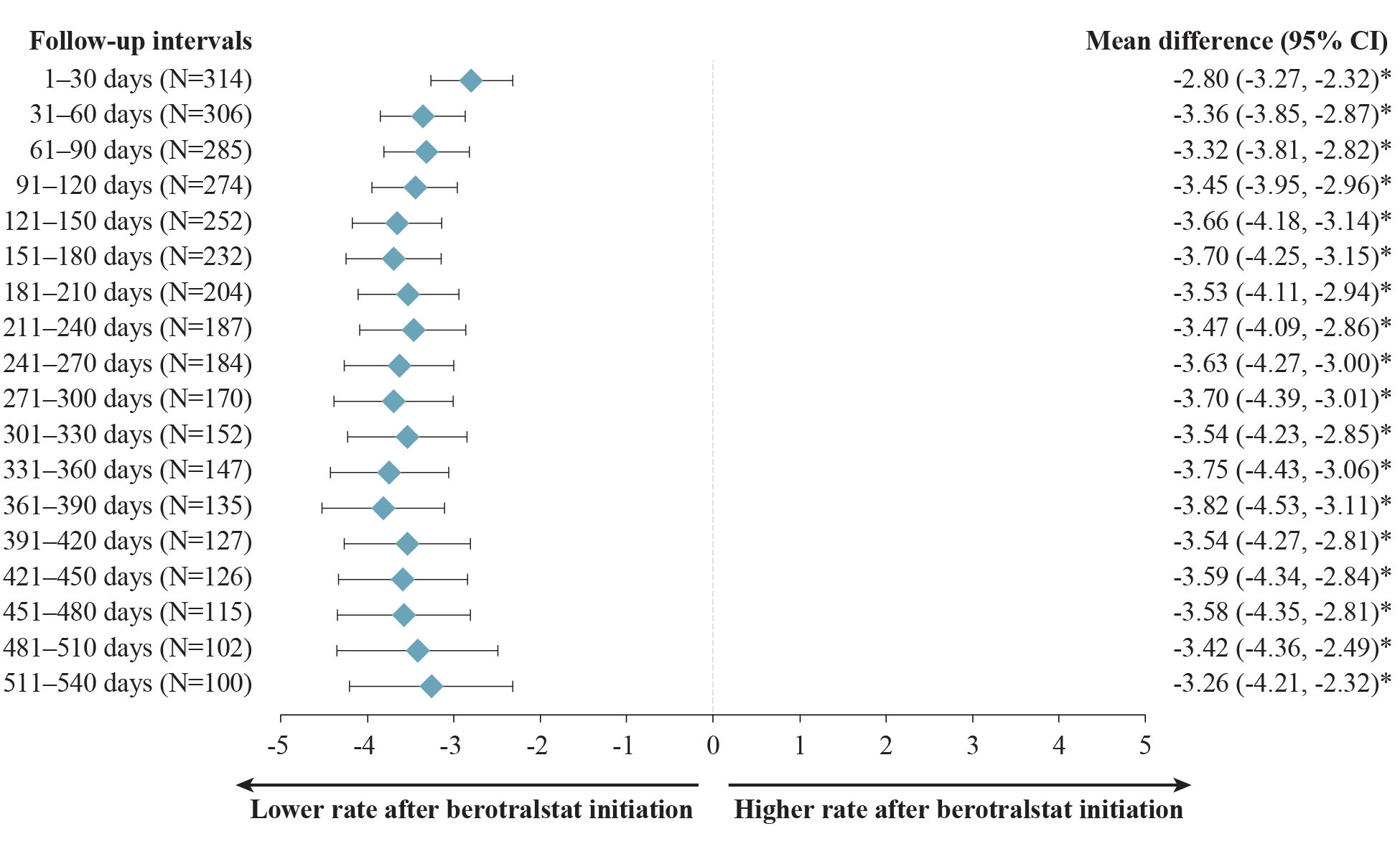


Abbreviations: CI, confidence interval; HAE-nC1INH, hereditary angioedema with normal C1 esterase inhibitor. **P*<.05.
